# The Cycad Genotoxin MAM Modulates Brain Cellular Pathways Involved in Neurodegenerative Disease and Cancer in a DNA Damage-Linked Manner

**DOI:** 10.1371/journal.pone.0020911

**Published:** 2011-06-23

**Authors:** Glen E. Kisby, Rebecca C. Fry, Michael R. Lasarev, Theodor K. Bammler, Richard P. Beyer, Mona Churchwell, Daniel R. Doerge, Lisiane B. Meira, Valerie S. Palmer, Ana-Luiza Ramos-Crawford, Xuefeng Ren, Robert C. Sullivan, Terrance J. Kavanagh, Leona D. Samson, Helmut Zarbl, Peter S. Spencer

**Affiliations:** 1 Center for Research on Occupational and Environmental Toxicology, Oregon Health & Science University, Portland, Oregon, United States of America; 2 Center for Environmental Health Sciences and Biological Engineering Division, Massachusetts Institute of Technology, Cambridge, Massachusetts, United States of America; 3 Department of Environmental and Occupational Health Sciences, Center for Ecogenetics and Environmental Health, University of Washington, Seattle, Washington, United States of America; 4 National Center for Toxicological Research, United States Food and Drug Administration, Jefferson, Arkansas, United States of America; 5 Center for Research on Occupational and Environmental Toxicology and Global Health Center, Oregon Health & Science University, Portland, Oregon, United States of America; 6 Department of Environmental and Occupational Health Sciences, University of Washington, Seattle, Washington, United States of America; 7 Division of Public Health Sciences, Fred Hutchinson Cancer Research Center, Seattle, Washington, United States of America; Brigham and Women's Hospital, Harvard Medical School, United States of America

## Abstract

Methylazoxymethanol (MAM), the genotoxic metabolite of the cycad azoxyglucoside cycasin, induces genetic alterations in bacteria, yeast, plants, insects and mammalian cells, but adult nerve cells are thought to be unaffected. We show that the brains of adult C57BL6 wild-type mice treated with a single systemic dose of MAM acetate display DNA damage (*O* ^6^-methyldeoxyguanosine lesions, *O* ^6^-mG) that remains constant up to 7 days post-treatment. By contrast, MAM-treated mice lacking a functional gene encoding the DNA repair enzyme *O* ^6^-mG DNA methyltransferase (MGMT) showed elevated *O* ^6^-mG DNA damage starting at 48 hours post-treatment. The DNA damage was linked to changes in the expression of genes in cell-signaling pathways associated with cancer, human neurodegenerative disease, and neurodevelopmental disorders. These data are consistent with the established developmental neurotoxic and carcinogenic properties of MAM in rodents. They also support the hypothesis that early-life exposure to MAM-glucoside (cycasin) has an etiological association with a declining, prototypical neurodegenerative disease seen in Guam, Japan, and New Guinea populations that formerly used the neurotoxic cycad plant for food or medicine, or both. These findings suggest environmental genotoxins, specifically MAM, target common pathways involved in neurodegeneration and cancer, the outcome depending on whether the cell can divide (cancer) or not (neurodegeneration). Exposure to MAM-related environmental genotoxins may have relevance to the etiology of related tauopathies, notably, Alzheimer's disease.

## Introduction

We describe mouse brain cell-signaling pathways that are perturbed by the aglycone (methylazoxymethanol, MAM) metabolite of a plant genotoxin (MAM-glucoside, cycasin) that is strongly associated with a declining neurodegenerative disease: Western Pacific amyotrophic lateral sclerosis and parkinsonism-dementia complex (ALS-PDC). This disease is clinically related to amyotrophic lateral sclerosis, atypical parkinsonism, and Alzheimer's dementia (AD) [1]–[6]. As with AD and certain other human neurodegenerative disorders, the cellular neuropathology of ALS-PDC is hallmarked by neurofibrillary tangles composed of paired helical filaments containing abnormally hyperphosphorylated forms of the microtubule-stabilizing protein tau [3], [6].

Western Pacific ALS-PDC, a prototypical neurodegenerative disorder apparently of environmental origin, has been highly prevalent in three genetically distinct island populations: (a) Japanese in the Kii Peninsula of Honshu Island, (b) Papuan New Guineans in West Papua, Indonesia, and (c) Chamorros on Guam and Rota in the Mariana Islands, migrants from Guam, and a few North American (Caucasian) and Filipino immigrants to Guam [6]–[8]. All three affected populations used the neurotoxic cycad seed for medicinal purposes [9]–[11]. On Guam, where ALS-PDC has been studied scientifically for over 60 years [4], the disease has been repeatedly linked to ingestion of food derived from cycad seed, with highly significant correlations for the cycasin content of cycad flour and for ALS and PD forms of the disease in both males and females [1], [5], [12], [13]. Diminishing use of cycad seed for food and/or medicine as affected populations adopt modern lifestyles is consistent with the progressive decline in disease prevalence in all three geographic isolates of ALS-PDC [11].

Cycasin and its aglycone methylazoxymethanol (MAM) are established developmental neurotoxins. MAM acetate damages neuronal DNA, modulates brain molecular networks and arrests regional brain development when administered systemically to postnatal day-3 mice [14], [15], but the adult rodent brain has been seen as largely refractory to the genotoxin [16], [17]. The promutagen MAM is also an established hepatotoxin and experimental carcinogen [18]. In fact, rodents that have been chronically treated with the MAM precursor azoxymethane (AOM) are widely used as models for investigating the pathogenesis and chemoprevention of human colon carcinoma [19]. Unfortunately, while recent (1998–2002) cancer data are available for Guam [20], longitudinal trends in cancer prevalence comparable to those available for Guam ALS-PDC are unknown.

We undertook this study of the adult mouse brain to test the hypothesis that the DNA-damaging properties of MAM, which are mutagenic and tumorigenic in cycling cells of the colon epithelium [19], activate molecular networks associated with the degeneration of post-mitotic neurons in neurodegenerative disease. While the relationship between environment-induced DNA damage, mutagenesis and malignancy is well accepted, non-nuclear mechanisms are usually considered to underpin neurodegenerative diseases. However, unlike most organs, the adult human brain has a low or absent capacity to repair alkylation-induced DNA damage [21], [22], with implications for long-term survival and eventual degeneration of nerve cells [23]. We addressed the aforementioned hypothesis by comparing the relationship between MAM-induced DNA damage (*O* ^6^-methyldeoxyguanosine, *O* ^6^-mG) and gene expression patterns in the brains of adult mice that are functionally proficient (wild type, wt) and deficient (*Mgmt^−/−^*) in the repair of *O* ^6^-mG, the latter lacking the gene coding for the specific DNA repair enzyme *O* ^6^-mG methyltransferase [24]. Two separate laboratories treated groups of wt and *Mgmt^−/−^* mice with a single systemic dose of MAM, and the combined data were mined for common brain transcriptional profiles. A third laboratory conducted blinded analyses of brain *O* ^6^-mG levels.

We present evidence that signaling pathways associated with human neurodegenerative disease are activated in mature mouse brain as the result of unrepaired MAM-induced DNA damage. These pathways involve receptors for certain neurotransmitters, including ionotropic and metabotropic neuronal receptors for glutamate, an excitatory neurotransmitter with the potential to kill nerve cells. While these findings support a role for MAM in the etiology of ALS-PDC, perhaps acting as a “slow toxin” via persistent DNA damage in nerve cells subject to continuous glutamate neurotransmission, they do not exclude a role for other cycad neurotoxins or genetic factors.

We also demonstrate that MAM-induced DNA damage modulates signaling pathways in the mouse brain that are associated with cancer, as well as neurodegeneration, the two phenotypes possibly representing responses of cycling and non-cycling cells, respectively. Others have proposed links between neurodegeneration/cancer and cell cycle regulation, DNA repair, response to oxidative stress [25], [26], aberrant wingless and proto-oncogene Int-1 (Wnt) signaling [27], glycogen synthase kinase 3 *beta* (GSK3β) regulation [28], modulation of tumor protein (TP53 or P53) expression [29], and perturbations of tau in AD and prostate cancer [30]. Chronic inflammation is another characteristic feature of both cancer and AD [31].

## Results

### Organ Response to MAM

**Figure 1:**
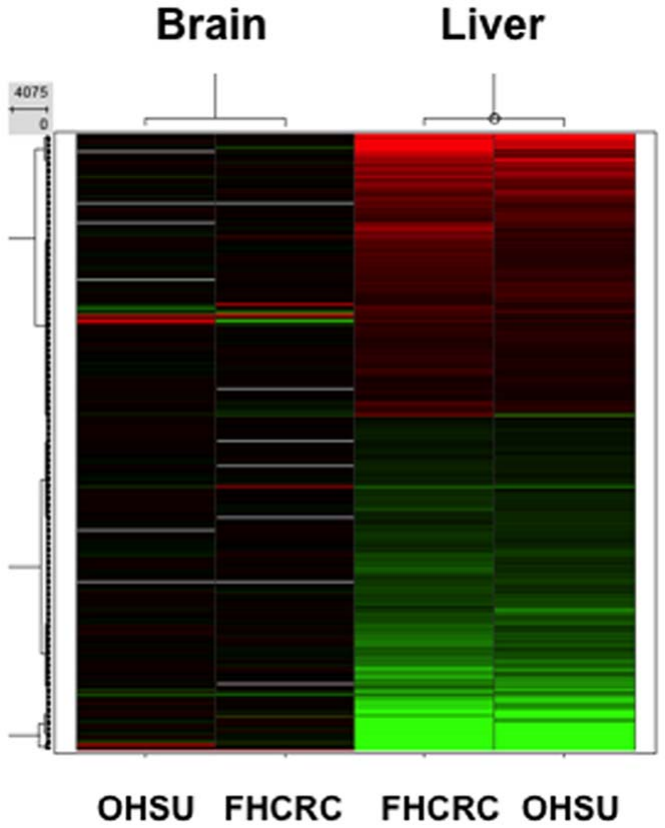
Heat map comparing the post-treatment (6 hr) transcriptional response of brain and liver (positive control) of wt mice to a single intraperitoneal dose of MAM. The experiment was conducted at two independent laboratories using identical protocols. Green denotes down-regulation and red up-regulation of gene expression. OHSU: Oregon Health & Science University. FHCRC: Fred Hutchinson Cancer Research Center.

A preliminary study was carried out to determine the immediate transcriptional responses of the mouse wt brain to systemic treatment with MAM relative to that of a non-neural tissue (liver) that is specifically targeted in humans with acute cycad toxicity. Comparable patterns of gene expression were found for the two tissues at the two study sites. Whereas the liver (a primary target of cycad toxicity in humans and rodents) showed a robust response to MAM, relatively few changes were noted in the brain (Fig. 1).

### DNA Damage

**Figure 2:**
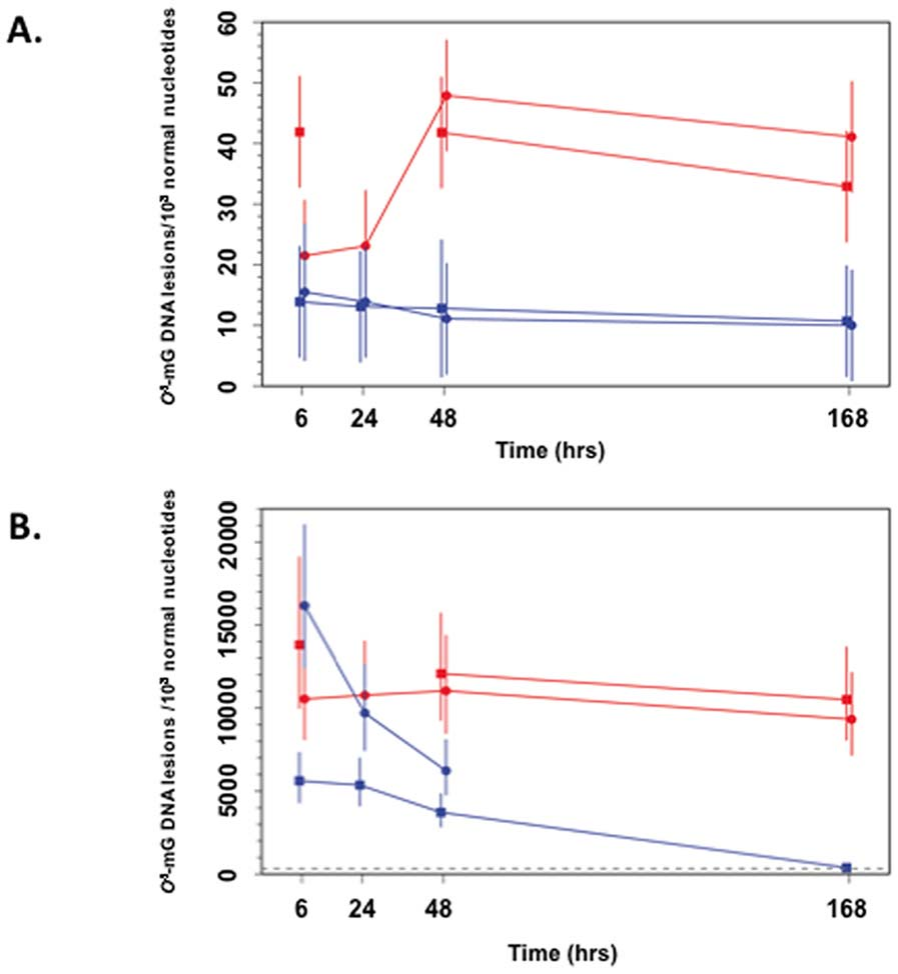
Time-course of *O^6^*-methylguanosine (*O^6^*-mG) DNA damage in the brain (A) and liver (B) of wt (blue) and *Mgmt^−/−^* (red) mice following a single intraperitoneal dose of MAM. Results for the two study sites are shown as separate red and blue lines. The plotting symbols (OHSU: circle; FHCRC: square) denote the estimated medians; lines extend ±2 standard errors from the medians. DNA damage (*O^6^*-mG) is three orders of magnitude higher in the liver than in brain, and the significantly elevated *O^6^*-mG levels in *Mgmt^−/−^* vs. wt tissues at 48 hr are maintained at 168 hr post MAM treatment. Discontinuities in the red and blue lines are attributed to technical errors or where samples were not collected. The dashed gray line in **B** denotes the maximum observed *O^6^*-mG level in the brain (∼330 lesions per 10^8^ normal nucleotides).

Brain tissue of wt and *Mgmt^−/−^* mice showed minimal detectable quantities (MDQ, see Methods) of *O^6^*-mG DNA lesions at 6 hr, 24 hr, 48 hr, and 168 hr following treatment with vehicle. After a single dose of MAM, the time-course of DNA damage in brain vs. liver (positive control) yielded fairly consistent data at the two independent study sites (Fig. 2). Levels of *O^6^*-mG were three orders of magnitude lower in brain than liver for both wt and *Mgmt^−/−^* mice. Significant deviation between the responses of wt and *Mgmt^−/−^* brain (*p<0.01*) and liver (*p<0.01*) was found at 48 hr, and this was maintained until 168 hr post MAM treatment (Table S1) [32]–[34]. While DNA damage was maintained at low (brain) or declining (liver) levels in the tissues of wt animals, there was persistence of the relatively higher levels of *O^6^*-mG DNA lesions in both tissues of *Mgmt^−/−^* mice. In sum, *O^6^*-mG lesions in both organs were much higher in *Mgmt^−/−^* vs. wt mice, and the DNA damage remained elevated in the brains of both wt and *Mgmt^−/−^* mice.

### Brain Transcriptional Profiles

Analyses were performed on data aggregated from two laboratories. No significant differences in gene expression were noted in the brains of wt mice treated with MAM vs. vehicle. By contrast, significant modulation of gene expression was present in the brains of similarly treated *Mgmt^−/−^* mice. Analyses were first performed to determine the transcriptional response to MAM in *Mgmt^−/−^* mice and, secondly, to explore the MAM vs. vehicle effect in each genotype and whether the modulation of the effect differed between the genotypes. Subsequently, Ingenuity® pathway analysis (IPA) was used to identify the most significantly enriched biofunctions for each data set by combining significant gene expression changes at all time-points and comparing these data. Gene expression changes were also anchored to *O^6^*-mG DNA lesions to determine which genes were modulated by DNA damage. A third analysis, which combined the first two analyses, was used to explore Kyoto Encyclopedia of Genes and Genomes (KEGG) pathways perturbed by MAM relative to vehicle that were either unique to *Mgmt^−/−^* or that differed between the two genotypes. The top KEGG pathways were determined using DAVID (the Database for Annotation, Visualization and Integrated Discovery) bioinformatics software [35], [36].

Initial studies were conducted to determine the response of the brain transcriptome to MAM vs. vehicle in DNA repair-deficient mice. The first set revealed 362 genes (of 41,000) that were differentially expressed in the brains of *Mgmt^−/−^* mice treated with MAM vs. vehicle (Table 1). Of these 362 genes, 57 were highly correlated (*r>0.7*) with *O* ^6^-mG levels. The four most significant disease biological functions corresponded to Neurological Disease (133 genes), Psychological Disorders (65 genes), Cancer (105 genes) and Genetic Disorder (170 genes). A list of the genes associated with each of these biological functions is provided in the supplemental data (Table S2).

**Table 1:** Top MAM-sensitive biological functions in *Mgmt^−/−^* mouse brains.

Diseases & Disorders	*Mgmt* ^−/−^ treated with MAM *vs.* vehicle (N = 362)	*Mgmt* ^−/−^ treated with MAM *vs.* vehicle (N = 57) anchored to *O* ^6^-mG
Neurological Disease	133	32
Psychological Disorders	65	19
Cancer	105	40
Genetic Disorder	170	23

Pathway analysis showing the four most significant biological functions altered in at least one time point in the brains of MAM-treated vs. vehicle-treated *Mgmt^−/−^* mice. The center column shows 362 genes that were significantly modulated by MAM, of which 57 genes individually satisfied criteria for DNA lesion anchoring (right).

**Figure 3:**
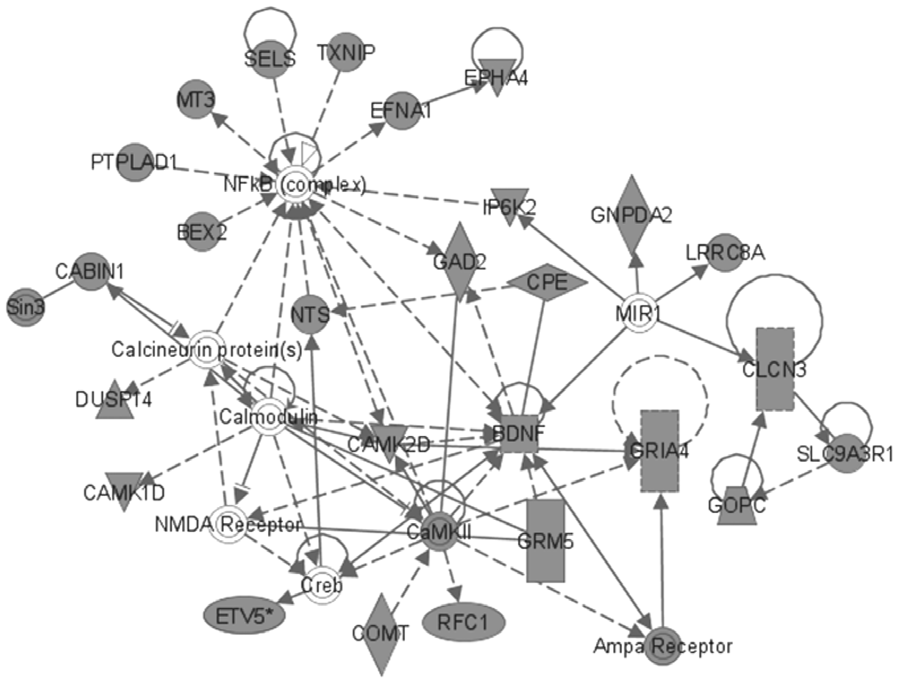
MAM-modulated brain gene products for *Mgmt^−/−^*. Most significant brain expression sub-network modulated by MAM vs. vehicle in *Mgmt^−/−^* mice (all time-points combined) composed of 362 differentially expressed genes.

**Figure 4:**
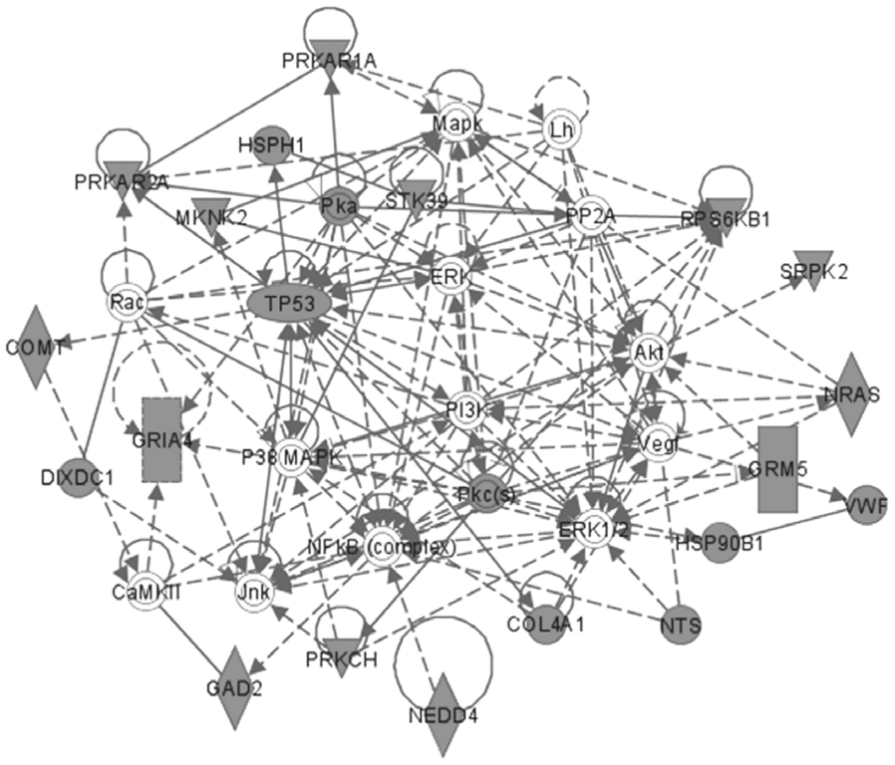
MAM-modulated brain gene products (anchored) for *Mgmt^−/−^*. Most significant brain expression sub-network modulated by MAM vs. vehicle in *Mgmt^−/−^* mice (all time-points combined) derived from 57 differentially expressed genes (a sub-set of the 362 genes) that were anchored to *O* ^6^-mG levels.

The most significant molecular networks derived from MAM-triggered, differentially expressed genes (362 genes) revealed hubs involving NF-κB (nuclear factor of *kappa* light polypeptide gene enhancer in B-cells), calcium-binding proteins (i.e., calcineurin, calmodulin), brain-derived neurotrophic factor (BDNF), glutamate receptors *N*-methyl-D-aspartate (NMDA) and alpha-amino-3-hydroxy-5-methyl-4-isoxazolepropionic acid (AMPA), CREB (cyclic AMP response element-binding), and microRNA1 (MIR1-1) (Fig. 3). When these MAM-induced differentially expressed genes were anchored to *O^6^*-mG levels, a subset of 57 genes revealed prominent hubs for NF-κB, extracellular signal-regulated kinase (ERK) and ERK1/2, p38-mitogen-activated protein kinase/c-Jun *N*-terminal kinases (MAPK/JNK), TP53, and Akt (v-akt murine thymoma viral oncogene homolog) (Fig. 4).

The second analysis explored the effect of MAM relative to vehicle between the two genotypes (wt vs. *Mgmt^−/−^*); thus, it determined whether DNA-repair capacity influences the response of the brain transcriptome to MAM. This analysis was sensitive to those genes that may have shown different directions of modulation between the genotypes, even if neither modulation was significant on its own. There were 153 differentially expressed genes reflecting genotype differences between wt and *Mgmt^−/−^* in the brain's response to MAM vs. vehicle. Of the 153 genes, 60 genes (∼40%) were anchored to *O* ^6^-mG levels. Brains of these animals showed the same four biological functions for disease and disorders as in Table 1, with the single exception that the category Psychological Disorders was not significant in the anchored group (Table 2). A list of the genes associated with each of these biological functions is provided in the supplemental data (Table S3).

**Table 2:** Top MAM-sensitive biological functions in wt and *Mgmt^−/−^* mouse brains.

Diseases & Disorders	wt *vs. Mgmt* ^−/−^ treated with respect to MAM vs. vehicle (N = 153)	wt *vs. Mgmt* ^−/−^ treated with respect to MAM vs. vehicle (N = 60) anchored to *O* ^6^-mG
Neurological Disease	63	28
Psychological Disorders	32	-
Cancer	22	2
Genetic Disorder	87	37

Pathway analysis showing the four most significant biological functions altered in the brains of *Mgmt^−/−^ vs.* wt mice treated with respect to MAM vs. vehicle (all time-points combined).

**Figure 5:**
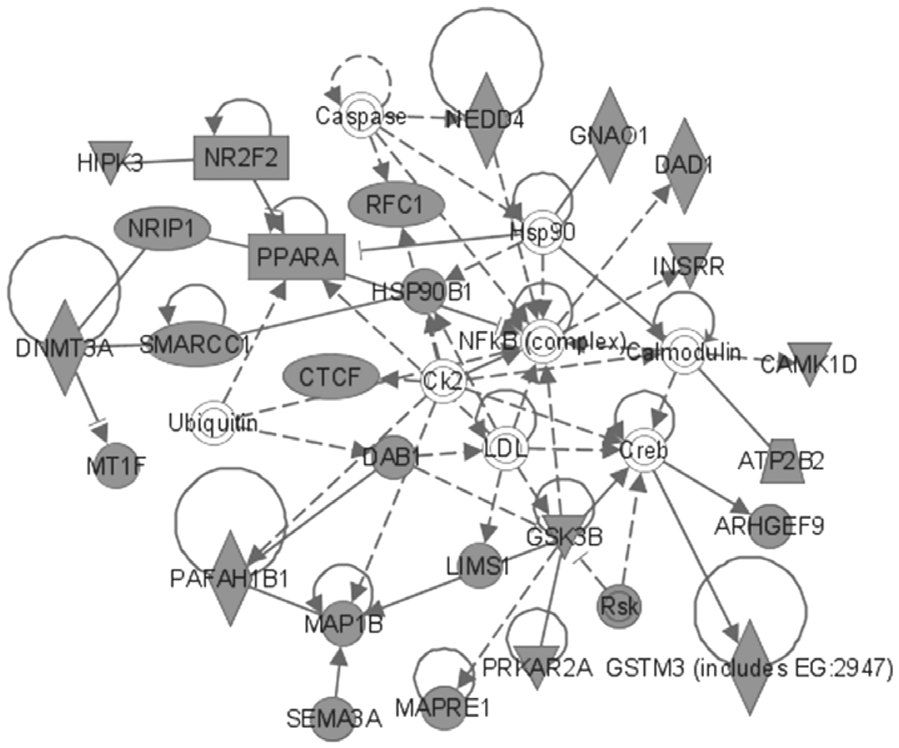
MAM-modulated brain gene products for *Mgmt^−/−^* vs. wild type. Most significant MAM-modulated expression sub-network in the brain of *Mgmt^−/−^ vs.* wild type mice (all time-points combined) composed of 153 differentially expressed genes. Note genes involved in epigenetic functions are also modulated: DNMT3A and SMARCC1 regulate chromatin function.

**Figure 6:**
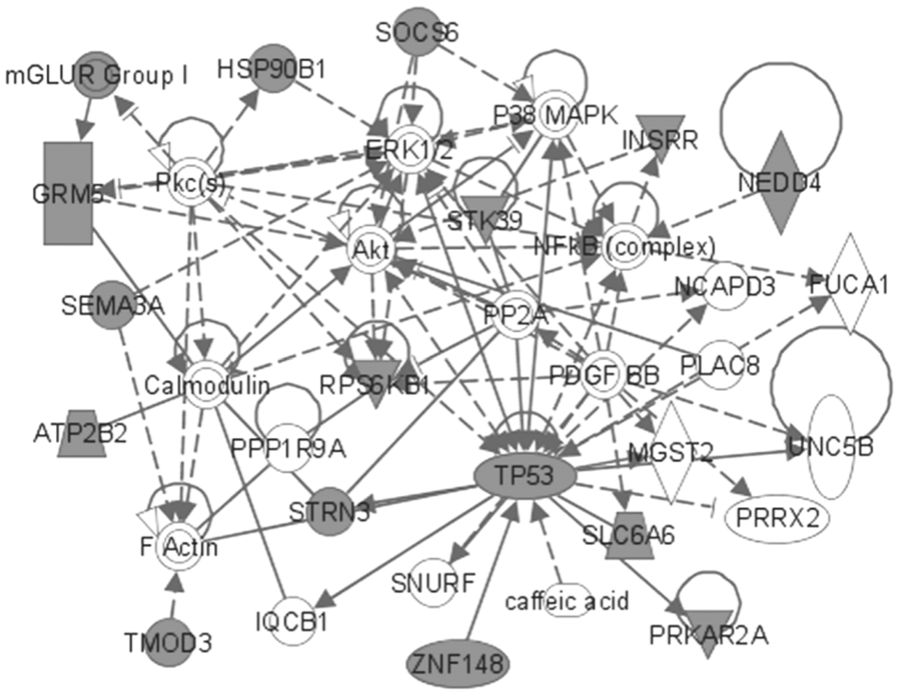
MAM-modulated brain gene products (anchored) for *Mgmt^−/−^* vs. wild type. Most significant MAM-modulated expression sub-network in the brain of *Mgmt^−/−^ vs.* wild type mice (all time-points combined) composed of 60 differentially expressed genes that were anchored to *O* ^6^-mG levels. Note the presence of NF-κB, ERK1, and p38-MAPK hubs, and the involvement of TP53 and glutamate receptors.

The most significant molecular networks in the 153 gene set contained hubs for NF-κB and CREB, and genes that regulate transcription through epigenetic mechanisms, including DNMT3A (DNA [cytosine-5-]-methyltransferase 3 *alpha*) and SMARCC1 (SWI/SNF related, Matrix-associated, actin-dependent regulator of chromatin, subfamily c, member 1) and nuclear transcription, namely PPARA (peroxisome proliferator-activated receptor *alpha*), molecular chaperones (HSP90B1, heat shock protein 90 kDa *beta* [Grp94], member 1), GSK3β and sema domain, immunoglobulin domain [Ig], short basic domain, secreted, [semaphorin] 3A (SEMA3A), which are implicated in Parkinson's and/or Alzheimer's disease (Fig. 5). As in Figure 3, the anchored set of 60 genes included TP53, ERK1/2 and NF-κB as the most prominent hubs, together with a number of other hubs (Akt, NF-κB, P38-MAPK and calmodulin) (Fig. 6). Glutamate receptors are also represented in this data set.

The third set of transcriptional data analyses combined the first two data sets (for a total of 443 non-duplicated genes), including the differential response of genotypes to MAM vs. vehicle (Table 2) and the response of *Mgmt^−/−^* brains to systemic treatment with MAM vs. vehicle (Table 1). The most significant scoring sub-network of MAM gene products (p<10^−46^) contained hubs for F-actin, NF-κB, microRNA-1, cofilin, calcium/calmodulin-dependent protein kinase II (CaMKII), glycogen synthase, the AMPA receptor, BDNF, and others. The same four diseases and disorders were the most significant of the biological functions list based on IPA analysis, while Nervous System Development and Function, and Skin and Hair Development and Function, appeared in the list of most significant four perturbed Physiological Systems Development and Functions (Table 3).

**Table 3:** Top MAM-associated diseases, disorders and physiological functions.

*Mgmt* ^−/−^ MAM vs. vehicle plus wt *vs. Mgmt* ^−/−^ with respect to MAM vs. vehicle (Total molecules N = 443)			
Diseases and Disorders		Physiological System Development and Function	
Neurological Disease	159	Nervous System Development & Function	64
Psychological Disorders	75	Embryonic Development	22
Cancer	114	Organ Development	14
Genetic Disorder	212	Skin and Hair Development and Function	11

Pathway analysis showing the four most significant diseases and disorders linked (left columns) and physiological systems (right columns) altered in the brains of MAM-treated vs. vehicle-treated *Mgmt^−/−^* mice *plus Mgmt^−/−^ vs.* wt mice treated with respect to MAM vs. vehicle. Data were aggregated and all time points were combined for a total of 443 molecules.

Table 4 shows the top KEGG pathways denoting molecular interactions perturbed by MAM in either wild type or *Mgmt^−/−^* brains. Pathways involved in cancer, Wnt signaling, and insulin-signaling pathways were among the most significant. Other prominent KEGG pathways included those involved in purine metabolism, MAPK signaling, neurotrophin signaling, chemokine signaling and neuroligand-receptor interaction (Table 4).

**Table 4:** Top MAM-associated brain KEGG pathways.

Top KEGG Pathways	Genes	Phenotype
Pathways in cancer	13	CC
Wnt signaling	10	AD, CC, skin, bone
Insulin signaling	9	AD, ALS
Purine metabolism	9	
Prostate cancer	8	CC
MAPK signaling	7	AD, CC
Melanogenesis	6	PD? CC
Neurotrophin signaling	6	AD
Focal adhesion	6	AD, CC
Chemokine signaling	5	AD
Neuroactive ligand-receptor interaction	5	AD
Calcium signaling pathway	4	AD, CC

Top brain KEGG pathways (derived from 443 MAM-modulated genes, see Table 3) and the number of MAM-modulated genes and human diseases associated with each pathway. Note the prominent involvement of the Wnt, insulin and MAPK signaling pathways, in addition to the presence of pathways involved in inflammatory and other responses. AD: Alzheimer disease. ALS: Amyotrophic lateral sclerosis. CC: Colon cancer. PD: Idiopathic Parkinson's disease.

**Figure 7:**
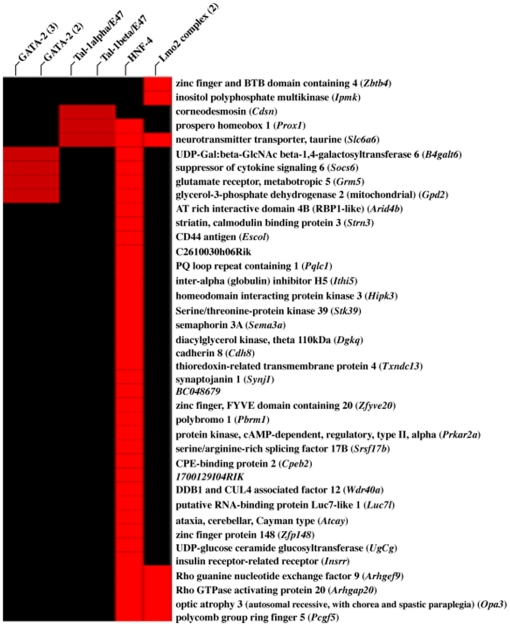
Transcription factor binding-site enrichment hierarchy. Analysis of the promoter regions of the 60-anchored gene sub-set derived from the strain-specific differentially expressed genes between MAM and vehicle-treated animals for transcription regulatory elements. A heat map (interaction matrix) shows the genes (rows) and motifs (columns) that were individually clustered and found in >5% of all promoters. Note the HNF4 binding site is common to 60% of the 60 anchored genes.

We also performed a PAINT (Promoter Analysis and Interaction Network Tool) analysis to identify the biologically relevant transcription factor binding sites in the genes that were enriched among the genotypes and differentially expressed genes between MAM and vehicle-treated animals. The 5′-flanking regions of the differentially expressed genes (2000 bp upstream of the transcription start site) were examined for enrichment of commonly expressed transcriptional regulatory elements (TREs). Table 5 shows the TREs among the unique genotype-specific (n = 153) and a subset of anchored (n = 60) genes targeted by MAM. Only TREs that were significantly enriched (p<0.05) in MAM-targeted genes are shown (Fig. 7). The highest scoring TRE was the highly conserved hepatocyte nuclear factor 4 (HNF-4), which binds to the consensus sequence AGGTCAaAGGTCA to activate transcription.

**Table 5:** MAM-associated enriched transcription factor binding sites.

Transcriptional Response Element	P Value
HNF-4	<0.001
Lmo-2	0.008
Tal-1α/E47	0.025
GATA-2	0.033
Tal-1β/E47	0.042

Enriched transcription factor binding sites among genotype-specific differentially expressed genes between MAM and vehicle-treated animals. Data based on 60 differentially expressed genes anchored to *O* ^6^-mG levels (see Fig. 6).

## Discussion

### Brain and Colon: Common Pathways but Different Outcomes

We have shown that the cycad genotoxin MAM induces persistent DNA damage (i.e., *O^6^*-mG lesions) and modulates several cell signaling pathways (i.e., TP53, NF-κB, MAPK) in the brains of young adult *Mgmt^−/−^* mice. Our data support the hypothesis that MAM-induced *O* ^6^-mG DNA lesions alter purine metabolism and modulate cell-signaling pathways associated with both neurodegeneration and cancer. While MAM does not induce brain tumors in singly treated adult mice, the genotoxin consistently triggers tumors in peripheral organs, notably the intestine [37], [38]. The prominent modulation of “cancer genes” in the “tumor-insensitive” brains of MAM-treated adult animals suggests that perturbations of these genes in the brain have consequences other than cancer.

**Figure 8:**
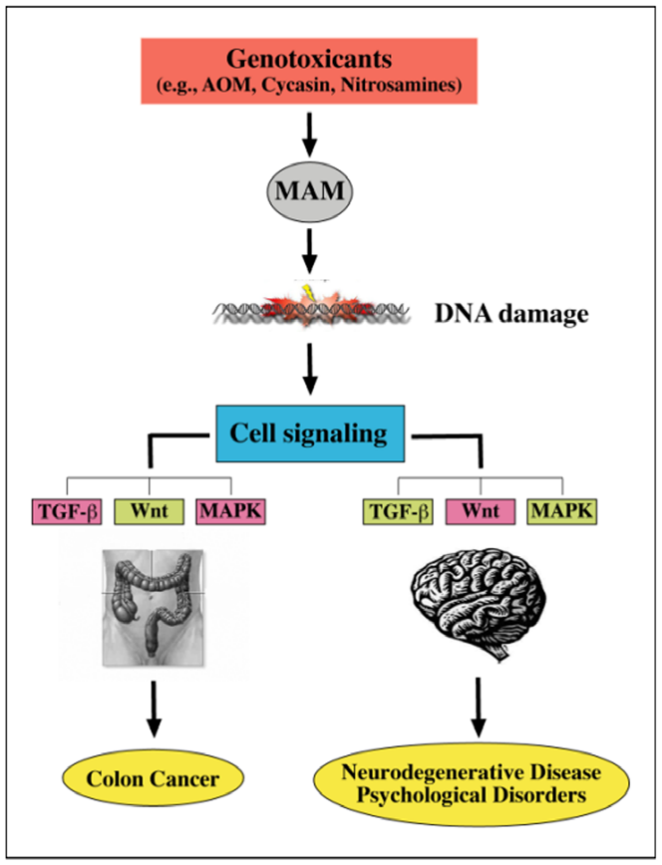
Proposed relationship between MAM-induced colon cancer and brain disease/disorders. Genotoxicants that induce *O^6^*-methylguanine lesions (DNA damage) (e.g. via methylazoxymethanol, MAM) disturb cell signaling pathways, including transforming growth factor-β (TGF-β), wingless and proto-oncogene Int-1 (Wnt), and mitogen-activated protein kinase (MAPK). In general, the literature supports up-regulation (green) and down-regulation (pink-red) in association with the two distinct phenoptypes. (Modified from Chen and Huang [40]).

Molecular mechanisms underlying MAM-induced colon cancer have been established in the azoxymethane (AOM) mouse model of colorectal adenocarcinoma in which MAM (the cytochrome P_450_2E1-mediated metabolite of AOM) is the sole triggering agent [39], [40]. In the AOM mouse model, MAM-induced mutation of *K-ras* (i.e., transversion from G:C to A:T at codon 12 derived from *O^6^*-mG lesions) activates this pathway and the downstream MAPK and Phosphoinositide 3-kinase/Akt (PI3K/Akt) mediators, indicating that MAM perturbs gene expression in these pathways by a DNA damage-dependent mechanism. Mutations in *β-catenin* blocks its degradation by a GSK-3β-mediated mechanism resulting in its intracellular accumulation and the activation of the Wnt/β-catenin signaling pathway. The nuclear transport of β-catenin leads to the activation of genes that regulate cell proliferation, while expression of pro-apoptotic proteins is inhibited [40], [41]. Such events may explain how MAM modulates the expression of genes with pivotal roles in cell signaling in the brain of young adult mice (Fig. 8).

### Cancer and Neurodegenerative Disease: Two Sides of the Same Coin?

The key finding relevant to ALS-PDC is the presence of MAM-induced transcriptional changes in the brains of young adult mice that lack efficient repair of *O* ^6^-methylguanine DNA lesions. This is in contrast to the absence of significant transcriptional changes in the adult brain of MAM-treated wild-type mice. The human brain shows variable amounts of MGMT activity, but most adult brains studied have minimal levels comparable to that of *Mgmt^−/−^* mice [21]. If the human brain responds to MAM in a manner comparable to the *Mgmt^−/−^* mouse brain, the response would be alterations in cell signaling pathways linked to both neurodegeneration and neuropsychological abnormalities. While the present results are based on short-term studies, the apparent association between the most significant MAM-associated biological functions in the adult mouse brain and cycasin-associated ALS-PDC are obvious. Their corollary provides clear support for further examination of the possible etiologic role of cycasin in the induction of ALS-PDC, one form (dementia) of which shows impressive clinical and neuropathological relationships with AD [42].

Behrens and colleagues [29] have reported an inverse relationship in the incidence of cancer and AD: in a prospective longitudinal study, the risk of developing cancer with time was significantly reduced in participants with AD, while those with a history of cancer had a lower rate of AD. The investigators state: “*in cancer, cell regulation mechanisms are disrupted with augmentation of cell survival and/or proliferation, whereas, conversely, AD is associated with increased neuronal death, either caused by, or concomitant with, β-amyloid (Aβ) and tau deposition*.” They discuss the putative role of P53 and the Wnt signaling pathway in these inverse disease associations: whereas reduced P53 expression arising from mutations may lead to uncontrolled cell proliferation, as, in colorectal cancer, bone cancer (osteosarcoma), and other tumors, increased P53 expression may activate pathways leading to cell death, such as occurs in AD [29]. The gene coding for TP53 was modulated by MAM in both DNA lesion-anchored sets of brain genes: while TP53 activation is known to occur after DNA damage, continued activation in the *Mgmt^−/−^* brain might promote neuronal demise.

### Links with Brain Pathology in ALS-PDC and AD

Wnt signaling and insulin signaling are also among the top KEGG pathways perturbed in the brain after systemic MAM treatment. While MAM-induced activation of the Wnt/β-catenin pathway leads to uncontrolled cell proliferation in the AOM model of colon cancer, suppression of this pathway in the brain may promote cell death. Boonen and colleagues [43] propose that disrupting the tightly regulated brain Wnt signaling pathway may constitute a key pathological event in AD. They propose that amyloid-*beta* (Aβ), a key protein in senile plaques, may down-regulate the Wnt/β-catenin pathway, thereby upregulating GSK3β and its subsequent hyperphosphorylation of tau, linking Aβ and neurofibrillary tangle pathology. Others have shown that inhibition of GSK3β increases mouse brain insulin-like growth factor-1 (IGF-1) [44], which in turn promotes Aβ production [45], [46]. IGF-1 and GSK3β are elevated in the hippocampus and spinal cord of individuals with Guam and Kii ALS [47]. IGF-1 is a potent survival factor for motor neurons in animals, and GSK3β is suspected to play important roles in apoptosis and tau phosphorylation [47].

The involvement of insulin signaling in AD has led to the proposal that this neurodegenerative disorder is a “special form of diabetes mellitus of the brain” [48]. The presence of microRNA1 as a differentially regulated hub in MAM-treated animals is noteworthy because of its ability to regulate insulin signaling (especially the IGF-1 receptor) [49], its association with colon cancer [50], and the key roles of ERK1 and microRNAs in tau phosphorylation and AD [51]. The insulin signaling pathway in diabetes mellitus type 2 is regulated by a number of transcription factors, notably HNFs, especially MODY1 (HNF4α), which regulates a large fraction of the hepatic and pancreatic transcriptomes by binding directly to approximately half of the transcribed genes. Therefore, HNF-4 serves as a “master regulator” of the human genome [52], [53]. There is substantial evidence that HNF-4 has a unique role in glucose-dependent insulin secretory pathways [54], [55], since mutations within the *HNF-4α* gene are linked to the monogenetic disorder Mature Onset Diabetes of the Young (MODY-1) [56]. HNF-4 was the most over-represented transcription factor-binding site among the promoter regions of brain genes that were modulated by MAM and anchored to DNA lesions. Since HNF-4 binds as a homodimer to a DNA recognition site containing a guanine-rich direct repeat element (AGGTCAaAGGTCA), this transcription factor might be a ‘hotspot’ for MAM-induced *O^6^*-mG DNA lesions. Several studies have shown that minor alterations in a nucleobase (e.g., *O^6^*-mG, 8-oxoG) at a crucial position within a promoter element can disrupt transcription factor binding and potentially modify gene expression [57]–[61]. Such a mechanism might explain why the HNF-4 consensus sequence was primarily targeted by MAM in the genes that were anchored to *O^6^*-mG DNA lesions.

The P38-MAPK signaling pathway is also among the top KEGG pathways perturbed by MAM. This cascade is activated following genotoxic stress [62], involved in the AOM model of colorectal cancer. and is widely believed to contribute to neuroinflammation in AD [63]. P38-MAPK has important roles in brain function, including glutamate (AMPA) receptor trafficking, NMDA-induced outward currents, excitotoxicity, synaptic plasticity and tau phosphorylation [63]–[66]. P38-MAPK hubs, linked to ionotropic and metabotropic glutamate receptors, were prominent in the anchored DNA damage data sets derived from the brains of MAM-treated mice suggesting that MAM-induced genotoxic stress perturbs glutamatergic function via a P38-MAPK-mediated mechanism. Given that MAM modulates glutamate-stimulated neuronal tau mRNA expression *in vitro* [67], we have proposed elsewhere that continuous MAM activation of glutamate-stimulated tau expression could trigger a slowly progressive neurodegenerative disease (tauopathy) of the type seen in Western Pacific ALS-PDC [68]. This mechanism is consistent with the observation that human exposure to cycad is followed by a clinically silent, long-latent period spanning years or decades [7], [13].

### Other links with ALS-PDC

There are other reasons to suspect an etiological relationship between the cycad genotoxins cycasin/MAM and Western Pacific ALS-PDC. First, treatment of postnatal mice with MAM arrests cerebellar development [69] leading to the production of ectopic, multinucleated Purkinje-like cells comparable to those reported in Guam and Kii ALS brains [70]. Second, individuals with ALS-PDC and cycad-exposed animals develop skin and bone changes (summarized by Spencer [71]). The Wnt signaling pathway, which was perturbed by MAM, plays a pivotal role in the regulation of bone mass, with pathway activation in bone regeneration [72], [73]. Wnt proteins (Wnt5a) also regulate epidermal differentiation in adult skin [74]. Animals grazing on cycads have a tendency to lose their horns and hooves, in addition to developing neuromuscular disease, and Guam Chamorros have had a high and familial incidence of benign bony nodules or multiple exostoses (diaphyseal aclasis) [71] and thickened skulls (unpublished data). Cycads speed skin repair in rodents, and the skin of ALS and ALS-PDC patients is uncommonly resistant to bed sores [68]. ALS skin shows increased expression of TDP-43 [75], one of the proteins that accumulates in ALS/PDC brains along with tau, Aβ, α-synuclein and ubiquitin [42].

There are important implications for human health if the association between cycasin/MAM and neurodegeneration is confirmed in longer-term studies. First and foremost, this would radically impact understanding of the etiology of sporadic ALS, AD and other tauopathies, and raise the intriguing possibility of creating animal models of these disorders using environmental agents that perturb genome regulation. Second, in the realm of public health, environmental agents and drugs with MAM-like genotoxic properties (e.g., nitrosamines, hydrazines, streptozotocin) would be suspect etiologic agents for [triggers of] sporadic neurodegenerative disease, particularly in individuals exposed early in life or those with impaired DNA-damage responses. Others have recently advanced the hypothesis that DNA-damaging nitrosamines, previously proposed as risk factors for cancer [76], may increase risk for AD as well as diabetes mellitus [77], [78]. Additionally, injection of the diabetogenic drug streptozotocin directly into the brain results in morphological abnormalities that include hyperphosphorylated tau and Aβ proteins [79]. These conditions, it should be noted, may involve long latent periods prior to clinical expression, consistent with the concept of a “slow toxin” proposed for cycasin [71].

### Summary

Given that Western Pacific ALS-PDC has occurred in genetically distinct populations that have used cycad for food and/or medicine, the present findings show that cycasin (the glucoside of MAM) is a plausible etiologic candidate for this disease. Single systemic treatment of adult mice with MAM rapidly damages brain DNA (*O^6^*-mG lesions) and modulates signaling pathways and neurotransmitter systems associated with human neurodegenerative disease. This statement is true only for animals with very low brain levels of the specific DNA-repair enzyme MGMT comparable to those in the young adult human brain. Murine brain signal pathways modulated by MAM and linked to human neurodegenerative disease overlap with those associated with MAM-induced colon cancer. The two disease phenotypes, neurodegeneration and cancer, are mechanistically related; their expression may depend on whether (colon epithelium) or not (neuron) MAM-exposed cells are able to undergo mitosis, mutagenesis and uncontrolled cell proliferation.

Our findings are based on the identification of interacting molecular networks, which permits identification of the key hubs for those networks. The hubs and the networks they connect, not the individual genes of each affected network, provide the key information in this study. Brain transcriptional changes linked to MAM-induced DNA damage were assessed within days of systemic treatment with the genotoxin to determine the earliest alterations in cellular function.

Finally, it is important to note that indictment of cycasin/MAM as a potential etiologic agent in ALS-PDC does not exclude a role for other cycad chemicals, including β-*N*-methylamino-L-alanine [12]; rather, it spurs the search for chemically related compounds that may play a role in sporadic forms of other tauopathies, including AD.

## Materials and Methods

### Ethics Statement

This study was carried out in strict accordance with the recommendations in the Guide for the Care and Use of Laboratory Animals of the National Institutes of Health (NIH). The protocol was approved by committees on the ethics of animal experimentation at the participating institutions. The Oregon Health & Science University (OHSU) Institutional Animal Care and Use Committee (IACUC) Protocols are #161 (G.E.K.) and B11100 (P.S.S.). The Fred Hutchinson Cancer Research Center (FHCRC) IACUC Protocol is #1595 (H.Z.).

### Mouse treatment with MAM

Methylazoxymethanol acetate (MAM purity ≥96% by GC) was purchased from Midwest Research Institute Chemical Carcinogen Repository (Kansas City, Missouri) and stored at −20°C. Eleven-week-old male C57BL6 wild type and *Mgmt* ^−/−^ mice on a C57BL6 background were acclimated for 5 days to local housing. *Mgmt* ^−/−^ mice were genotyped as previously described [24]. Animals were randomly assigned to two treatment groups (n = 4 per group) and given a single intraperitoneal injection (100 µl adjusted to body weight) with either vehicle (0.05% high-performance liquid chromatography-grade acetic acid in saline) or MAM (20 mg/kg in vehicle). Animals were terminated by guillotine 6 hr, 24 hr, 48 hr or 7 days post-injection, periods during which no signs of evolving neurological or behavioral changes were observed. Brains were rapidly excised and transected longitudinally: the right half was placed in ice-cold RNALater™ (Applied Biosystems/Ambion Inc, Austin, Texas) for transcriptional analysis and the left half flash-frozen for analysis of DNA lesions. This protocol was carried out in two independent laboratories (FHCRC, OHSU).

### DNA lesions

A blind assay for DNA damage in liver and individual half-brains was carried out at the Food & Drug Administration National Center for Toxicological Research (Jefferson, Arkansas) using a liquid chromatography tandem mass spectrometry (LC/MS-MS) procedure specific for *O^6^*-methyldeoxyguanosine DNA lesions [80]. Genomic DNA was isolated using Qiagen Genomic-tip 100/G columns, as described by the manufacturer (Qiagen, Valencia, California). Briefly, genomic DNA was purified by mechanically disrupting the tissue in lysis buffer with a Potter-Elvehjem homogenizer, the homogenate incubated with proteinase K, then washed twice with 70% ethanol, air-dried, and dissolved in LC-MS buffer (5 mM bis-Tris pH 7.1, 0.1 mM ethylenediaminetetraacetic acid). Purity was checked by 260/280 ratios (range 1.8–2.0), and the concentration determined by the NanoDrop™ method (NanoDrop Technologies, Wilmington, Delaware). An aliquot of genomic DNA (∼10–20 µg) was incubated with 4 units of micrococcal nuclease and 0.5 units of spleen phosphodiesterase overnight at 37°C in hydrolysis buffer (14 mM succinic acid, 8.5 mM calcium chloride, pH 6). Nuclease P1 (1.5 units in 1 mM zinc chloride) was added, the samples incubated at 37°C for 2 h, an internal standard added (^15^N-*O* ^6^-methyldeoxyguanosine) to the digest, and the samples centrifuged before analysis by LC/MS-MS. Reference DNA (calf thymus DNA treated with methyl nitrosourea to give ∼250 lesions in 10^7^ normal nucleotides) was used in each run to control for day-to-day variability. The minimal detectable quantity of DNA lesions in liver and brain tissue was 1–2.6 *O^6^*-mG lesions/10^8^ normal nucleotides.

The liver and brain of vehicle-treated wild type and *Mgmt^−/−^* mice showed minimal detectable quantities (MDQ = 1–2.6) of *O* ^6^-mG DNA lesions/10^8^ normal nucleotides at 6 hr, 24 hr, 48 hr and 168 hr post MAM treatment.

### Microarray processing and analysis

RNA was extracted from half-brains using the RNeasy Mini Kit (Qiagen, Valencia, California) and analyzed for concentration and quality with the Agilent Bioanalyzer (Agilent, Andover, Massachusetts). Three of the four RNA samples from each treatment group at each time point were frozen and shipped on dry ice to Icoria, Inc. (Research Triangle Park, North Carolina) where they were hybridized. Total RNA was labeled using the one-cycle target labeling protocol and hybridized to GeneChip® Mouse Genome 430 2.0 Arrays (Affymetrix, Santa Clara, California). Preliminary analysis of brain and liver data was performed by the Rosetta Resolver® system (Rosetta Biosoftware, Seattle, Washington) for gene expression analysis.

Data from raw cell intensity files (.CEL) were pre-processed and normalized using robust multi-array average (RMA). RMA values were calculated with R/Bioconductor's package [81]–[84] using the default arguments (background correction using RMA background correction, quantile normalization, and median polish). Data from each of the four time points were analyzed separately. A linear model was separately fitted to RMA-normalized intensity measures for each of the ∼45,000 probes. Models included terms for the three effects of interest: treatment (MAM vs. vehicle), genotype (wt vs. *Mgmt^−/−^*), and the interaction between treatment and genotype. Four additional model terms were included to account for the two laboratories (FHCRC vs. OHSU), and the interaction of laboratory with the three effects of interest. Consistency of these effects was assessed through the interactions involving the laboratory: if p>0.15 for interactions involving the laboratory, the initial model was reduced (by excluding interactions with laboratory) and then refitted and effects of interest estimated; if interactions with the laboratory were significant (p<0.15), then the probe was excluded from further analysis as this indicated effects of genotype, treatment, or the interaction between the two effects varied between laboratories. Probes with consistent effects between laboratories had *p*-values for treatment, genotype, and the interaction adjusted to control the false discovery rate (FDR) [85]. Probes were declared significant if the FDR-adjusted *p*-value was <0.05 and the absolute fold change for the particular effect was >1.3.

All data are compliant with the MIAME (Minimum Information About a Microarray Experiment standard. Raw data were deposited in Gene Expression Omnibus (GEO), using the GEO Accession Number GSE26600. http://www.ncbi.nlm.nih.gov/geo/query/acc.cgi?token=rxmhhmkgwmiwedq&acc=GSE26600.

### Anchoring Microarray Data to DNA lesions

For MAM-treated animals (wt and *Mgmt^−/−^*), Spearman's rank correlation coefficient was computed between RMA-normalized signal intensities and the number of *O^6^*-mG lesions. Correlations were computed separately for each time point. Correlation coefficients between lesion levels and normalized intensity >0.70 were taken as an indication of “anchoring” between individual gene expression and brain DNA damage. No significant change in brain gene expression was found in wt mice treated with MAM.

### Network analysis

Probes that showed significance in the microarray analysis for at least one time point (6 h, 24 h, 48 h or 7 days post-injection) were analyzed for enriched networks using Ingenuity® software (http://www.ingenuity.com/). Networks were developed separately for probe sets where the MAM vs. vehicle effect was modified by genotype and also for probes where the MAM vs. vehicle effect was only significant in the *Mgmt^−/−^* mice. Network analysis was further sharpened by restricting the focus to probes anchored to DNA lesion levels.

### Transcription Factor Binding-site Analysis

Transcription factor binding-site analysis was performed using PAINT (http://www.dbi.tju.edu/dbi/tools/paint/) [86]. PAINT identifies transcriptional response elements (TRE) in the 5′-untranslated regions of genes and assesses which TREs occur at a frequency different than expected by chance. p<0.05 was considered statistically significant. 5 kb of the 5′-untranslated regions of the genomic sequences were used for the analysis. Default settings were used for all other parameter choices.

## Supporting Information

Table S1 **Median number of** ***O*** **^6^-mG DNA lesions for wt and** ***Mgmt^−/−^*** **animals.** Analysis of mouse brain DNA adduct levels using robust linear models (RLM, Wilcoxon weights) [32]–[34]. Data obtained from brains 6 hr post-systemic treatment with MAM. Median response (*O* ^6^-mG DNA lesions per 10^8^ normal nucleotides) given along with estimated standard error of median in square brackets. Tests compare median number of *O* ^6^-mG DNA lesions per 10^8^ normal nucleotides between *Mgmt^−/−^* and wild type mice; reported test statistic (TS) is distributed approximately as F(1,28).
(DOCX)

Table S2 **List of genes supporting data in** **Table 1** **. Genes modulated by MAM within each of the four top biological functions (MAM vs. vehicle).**
(DOC)

Table S3 **List of genes supporting data in** **Table 2** **. Genes modulated by MAM within each of the four top Biological Functions (wt vs.** ***Mgmt*** **^−/−^).**
(DOC)
