# Supplementary material for: The Cycad Genotoxin MAM Modulates Brain Cellular Pathways Involved in Neurodegenerative Disease and Cancer in a DNA Damage-Linked Manner
Source: PLoS One. 2011 Jun 23;6(6):e20911. doi: 10.1371/journal.pone.0020911 (PMC3121718; doi:10.1371/journal.pone.0020911)
Supplement: Table S1 — Median number of O 6-mG DNA lesions for wt and Mgmt−/− animals. Analysis of mouse brain DNA adduct levels using robust linear models (RLM, Wilcoxon weights) [32]–[34]. Data obtained from brains 6 hr post-systemic treatment with MAM. Median response (O 6-mG DNA lesions per 108 normal nucleotides) given along with estimated standard error of median in square brackets. Tests compare median number of O 6-mG DNA lesions per 108 normal nucleotides between Mgmt−/− and wild type mice; reported test statistic (TS) is distributed approximately as F(1,28). (DOCX) [file pone.0020911.s001.docx]

|  |  | Time (hours) | | | |
| --- | --- | --- | --- | --- | --- |
| Laboratory | Genotype/Test | 6 | 24 | 48 | 168 |
| FHCRC | *Mgmt ^-/-^* | 41.9 [4.55] | ---* | 41.8 [4.55] | 32.9 [4.55] |
|  | Wild type (wt) | 13.9 [4.55] | 13.1 [4.55] | 12.8 [5.63 | 10.7 [4.55] |
|  | *Mgmt ^-/-^* vs wt | TS=19.6, p<0.001 | ---* | TS=16.5, p<0.001 | TS=7.32, p<0.001 |
| OHSU | *Mgmt ^-/-^* | 21.5 [4.56] | 23.1 [4.55] | 47.9 [4.55] | 41.1 [4.55] |
|  | Wild type (wt) | 15.5 [5.63] | 13.9 [4.55] | 11.1 [4.55] | 10.0 [4.55] |
|  | *Mgmt ^-/-^* vs wt | TS=0.50, p=0.484 | TS=2.99, p=0.095 | TS=16.5, p<0.001 | TS=23.1, p<0.001 |

* all three samples contained no detectable adducts, suggesting erroneous samples
