# Supplementary material for: The Cycad Genotoxin MAM Modulates Brain Cellular Pathways Involved in Neurodegenerative Disease and Cancer in a DNA Damage-Linked Manner
Source: PLoS One. 2011 Jun 23;6(6):e20911. doi: 10.1371/journal.pone.0020911 (PMC3121718; doi:10.1371/journal.pone.0020911)
Supplement: Table S2 — List of genes supporting data in Table 1 . Genes modulated by MAM within each of the four top biological functions (MAM vs. vehicle). (DOC) [file pone.0020911.s002.doc]

| **Neurological Disease** | **Psychological Disorders** | **Cancer** | **Genetic Disorder** |
| --- | --- | --- | --- |
| KCND2 | KCND2 | MALAT1 | MALAT1 |
| CAB39 | CAMK1D | FTL | CAB39 |
| TPD52L2 | DLG2 | TPD52L2 | KCND2 |
| FTL | FAM115A | AHCYL1 | FTL |
| CREM | ELAVL4 | CPEB2 | TPD52L2 |
| AHCYL1 | DAB1 | DLG2 | CREM |
| ETHE1 | GRIA4 | LIMS1 | AHCYL1 |
| DLG2 | GLO1 | BEX2 | DLG2 |
| FAM115A | CABIN1 | GRIA4 | ETHE1 |
| ELAVL4 | GAD2 | GLO1 | FAM115A |
| DAB1 | CAMK2D | GAD2 | ELAVL4 |
| HRNBP3 | COMT | HOOK3 | DAB1 |
| GRIA4 | FABP7 | MERTK | GRIA4 |
| GLO1 | CDH13 | SMG1 | HRNBP3 |
| CABIN1 | KCNA6 | FABP7 | GLO1 |
| GAD2 | ZFHX3 | CDH13 | CABIN1 |
| PRPS1 | TP53 | ZFHX3 | SRPK2 |
| MERTK | ATG5 (includes EG:9474) | TNFRSF21 | GAD2 |
| FABP7 | STK39 | TP53 | PRPS1 |
| CDH13 | LPHN3 | CKS2 | MERTK |
| ZFHX3 | ANKHD1 | HBA1 | GPD2 |
| TNFRSF21 | KLF13 | NEDD4 | FABP7 |
| TP53 | NEDD4 | ANP32A | CDH13 |
| ATG5 (includes EG:9474) | NAPG | RAB31 | ZFHX3 |
| LPHN3 | CCND2 | AKAP13 | TP53 |
| AGXT2L1 | ITIH5 | TIMP4 | ATG5 (includes EG:9474) |
| KLF13 | UQCRC2 | CCND2 | CKS2 |
| NEDD4 | ZCCHC24 | ARAF | LPHN3 |
| ANP32A | PBRM1 | ACP1 | HBA1 |
| OPA3 | TCF4 | MBD4 | AGXT2L1 |
| RAB31 | OSBPL6 | SPINT2 | KLF13 |
| AKAP13 | BDNF | ALCAM | NEDD4 |
| CCND2 | SRD5A1 | CANT1 | ANP32A |
| ARAF | MAPT | TCF4 | OPA3 |
| ACP1 | PDE1A | PDE7A | RAB31 |
| UQCRC2 | ABLIM1 | SRD5A1 | AKAP13 |
| MBD4 | GABRB2 | BDNF | TIMP4 |
| ALCAM | PHYHD1 | SOCS2 | CCND2 |
| ATOX1 | HSP90B1 | STRA13 | ARAF |
| ALAS2 | NFKBIA | EPHA4 | ACP1 |
| TCF4 | COQ7 | EIF4G1 | UQCRC2 |
| BDNF | PCMT1 | POLD4 | MBD4 |
| SRD5A1 | CIT | DCLK1 | FAM13C |
| EPHA4 | ZBTB4 | MXD1 | ALCAM |
| EIF4G1 | DGCR8 | DGCR8 | ALAS2 |
| PRKG1 | PITPNC1 | ZBTB4 | TCF4 |
| ABLIM1 | GRIK2 | RB1CC1 | PDE7A |
| CORO2B | GATM | CTNNB1 | SRD5A1 |
| PHYHD1 | NELL2 | TXNIP | BDNF |
| DCLK1 | HIPK3 | EFNA1 | STRA13 |
| OGT | TXNIP | ATP2B2 | EPHA4 |
| DGCR8 | HSPH1 | CEACAM1 | POLD4 |
| MXD1 | MT3 | ZNF148 | CORO2B |
| ZBTB4 | DAAM2 | RNF5 (includes EG:6048) | ABLIM1 |
| KIAA1267 | GABRP | PEX5 | PRKG1 |
| RB1CC1 | ATP2B2 | GMPS | PHYHD1 |
| CELF4 | GRM5 | BCL11A | DCLK1 |
| GRIK2 | ALB | EIF4A1 | OGT |
| CTNNB1 | EBF1 | NRIP1 | AMN |
| NAPB | LIN7B | SLC5A5 | MXD1 |
| NELL2 | GMPS | PTPRT | ZBTB4 |
| TXNIP | SYNJ1 | CELF1 | DGCR8 |
| SLC6A6 | KIF5B | TP53RK (includes EG:112858) | RB1CC1 |
| FKTN | PTPRT | ZNF703 | KIAA1267 |
| ID3 | PRKAR1A | COMT | CELF4 |
| ATP2B2 |  | SMYD4 | GRIK2 |
| GRM5 |  | HBD | CTNNB1 |
| RNF5 (includes EG:6048) |  | JUP | NAPB |
| B4GALT6 |  | CLCN3 | NELL2 |
| PEX5 |  | PDK4 | TXNIP |
| GMPS |  | RPS6KB1 | SLC6A6 |
| PTPRT |  | COL4A1 | FKTN |
| CAMK1D |  | TTC3 | CSGALNACT1 |
| IGBP1 |  | ITIH5 | ID3 |
| CAMK2D |  | PRKCH | GNPTG |
| COMT |  | PSMD4 | GIT2 |
| CLCN3 |  | RBBP4 | ATP2B2 |
| MYO10 |  | ETV5 | ZNF148 |
| DEPDC6 (includes EG:64798) | | TPM1 | GRM5 |
| RPS6KB1 |  | NLGN1 | CEACAM1 |
| COL4A1 |  | MAPT | TNS3 |
| GJC2 |  | NTS | RNF5 (includes EG:6048) |
| ICA1 |  | UGCG | B4GALT6 |
| STK39 |  | SLC2A3 | PEX5 |
| RDX |  | GABRB2 | GMPS |
| ANKHD1 |  | HSP90B1 | BCL11A |
| NAPG |  | SOX9 | EIF4A1 |
| TTC3 |  | POLR2A | NRIP1 |
| LMCD1 |  | NFKBIA | SLC5A5 |
| ITIH5 |  | RGS5 | PTPRT |
| CDH8 |  | ARL5B | CAMK1D |
| PRKCH |  | IGHM | DNMT3A |
| CPE |  | ARFGEF1 | IGBP1 |
| ZCCHC24 |  | PXN | ESF1 |
| ETV5 |  | NRAS | C8ORF79 |
| PBRM1 |  | SLC9A3R1 | CAMK2D |
| OSBPL6 |  | ATRX | COMT |
| NLGN1 |  | CSDE1 | ARHGAP20 |
| MARK2 |  | VWF | HBD |
| MAPT |  | XPA | JUP |
| UGCG |  | GABRP | CLCN3 |
| PDE1A |  | PGLYRP1 | MYO10 |
| SLC2A3 |  | ALB | DEPDC6 (includes EG:64798) |
| GABRB2 |  | CKS1B | RPS6KB1 |
| HSP90B1 |  | PRKAR1A | TSPAN5 |
| SOX9 |  |  | COL4A1 |
| NFKBIA |  |  | GJC2 |
| COQ7 |  |  | ICA1 |
| PCMT1 |  |  | STK39 |
| CIT |  |  | RDX |
| ITSN2 |  |  | ANKHD1 |
| PITPNC1 |  |  | NAPG |
| IGHM |  |  | WAC |
| PHF21A |  |  | LMCD1 |
| GATM |  |  | TTC3 |
| CNNM1 |  |  | ITIH5 |
| PXN |  |  | CDH8 |
| SLC9A3R1 |  |  | PRKCH |
| HIPK3 |  |  | PSMD4 |
| HSPH1 |  |  | CPE |
| ATRX |  |  | ZCCHC24 |
| PRKAR2A |  |  | ANKS1B |
| VWF |  |  | ETV5 |
| MT3 |  |  | PBRM1 |
| DAAM2 |  |  | TPM1 |
| XPA |  |  | OSBPL6 |
| GABRP |  |  | NLGN1 |
| ALB |  |  | MAPT |
| LIN7B |  |  | NTS |
| EBF1 |  |  | UGCG |
| SYNJ1 |  |  | INPPL1 |
| KIF5B |  |  | PDE1A |
| PRKAR1A |  |  | SLC2A3 |
|  |  |  | GABRB2 |
|  |  |  | SOX9 |
|  |  |  | HSP90B1 |
|  |  |  | NFKBIA |
|  |  |  | POLR2A |
|  |  |  | COQ7 |
|  |  |  | RGS5 |
|  |  |  | PCMT1 |
|  |  |  | CIT |
|  |  |  | ITSN2 |
|  |  |  | IGHM |
|  |  |  | PITPNC1 |
|  |  |  | LRRC8A |
|  |  |  | GATM |
|  |  |  | PHF21A |
|  |  |  | CNNM1 |
|  |  |  | NRAS |
|  |  |  | HIPK3 |
|  |  |  | SLC9A3R1 |
|  |  |  | HSPH1 |
|  |  |  | ATRX |
|  |  |  | HPS1 |
|  |  |  | PRKAR2A |
|  |  |  | DAAM2 |
|  |  |  | VWF |
|  |  |  | MT3 |
|  |  |  | XPA |
|  |  |  | ZRANB1 |
|  |  |  | GABRP |
|  |  |  | LIN7B |
|  |  |  | EBF1 |
|  |  |  | ALB |
|  |  |  | SYNJ1 |
|  |  |  | KIF5B |
|  |  |  | ZNF622 |
|  |  |  | CKS1B |
|  |  |  | PRKAR1A |
|  |  |  |  |
