# Supplementary material for: The Cycad Genotoxin MAM Modulates Brain Cellular Pathways Involved in Neurodegenerative Disease and Cancer in a DNA Damage-Linked Manner
Source: PLoS One. 2011 Jun 23;6(6):e20911. doi: 10.1371/journal.pone.0020911 (PMC3121718; doi:10.1371/journal.pone.0020911)
Supplement: Table S3 — List of genes supporting data in Table 2 . Genes modulated by MAM within each of the four top Biological Functions (wt vs. Mgmt −/−). (DOC) [file pone.0020911.s003.doc]

| **Neurological Disease** | **Psychological Disorders** | **Cancer** | **Genetic Disorder** |
| --- | --- | --- | --- |
| PPARA | PBRM1 | PPARA | PPARA |
| CAMK1D | PPARA | TP53 | CAMK1D |
| PDE3A | CAMK1D | RPS6KB1 | DNMT3A |
| MAP1B | PDE3A | CPEB2 | PDE3A |
| FAM115A | FAM115A | C19ORF10 | MAP1B |
| DAB1 | PDE1A | PDE3A | SOCS6 |
| HRNBP3 | DAB1 | GSTM3 (includes EG:2947) | NPEPPS |
| TIPARP | ABLIM1 | HESX1 | FAM115A |
| UBXN4 | HSP90B1 | CSDE1 | DAB1 |
| MPHOSPH9 | COQ7 | PROX1 | HRNBP3 |
| MTMR2 | PDLIM5 | LIMS1 | TIPARP |
| GSK3B | ZBTB4 | XPA | GPR123 |
| SUSD3 | CIT | ATP2B2 | IMPDH2 |
| TP53 | CLMN | HSP90B1 | UBXN4 |
| TNFRSF21 | EYA4 | RNF5 (includes EG:6048) | GPD2 |
| RPS6KB1 | GSK3B | IMPDH2 | ARHGAP20 |
| ATG5 (includes EG:9474) | TP53 | ITIH5 | MPHOSPH9 |
| STK39 | ATG5 (includes EG:9474) | ZBTB4 | MTMR2 |
| AGXT2L1 | HIPK3 | EIF4A1 | GSK3B |
| HESX1 | NELL2 | SMG1 | DCAF12 |
| CDSN | STK39 | GSK3B | SUSD3 |
| SUSD4 | SUSD4 | MT1F | PELI2 |
| GNG3 | KLF13 |  | TP53 |
| KLF13 | NEDD4 |  | RPS6KB1 |
| NEDD4 | ATP2B2 |  | ATG5 (includes EG:9474) |
| OPA3 | GRM5 |  | STK39 |
| UQCRC2 | SEMA3A |  | HESX1 |
| GNAO1 | SYNJ1 |  | AGXT2L1 |
| ITIH5 | BBX |  | SUSD4 |
| CDH8 | ITIH5 |  | CDSN |
| TRPM1 | UQCRC2 |  | GNG3 |
| ARHGEF9 | MT1F |  | KLF13 |
| MT1F |  |  | NEDD4 |
| PBRM1 |  |  | OPA3 |
| UGCG |  |  | GNAO1 |
| DDX3X |  |  | UQCRC2 |
| PDE1A |  |  | ITIH5 |
| ABLIM1 |  |  | CDH8 |
| HSP90B1 |  |  | TRPM1 |
| COQ7 |  |  | FAM13C |
| PDLIM5 |  |  | SCAPER |
| ZBTB4 |  |  | ARHGEF9 |
| CIT |  |  | PCGF5 |
| CLMN |  |  | MT1F |
| EYA4 |  |  | ANKS1B |
| NAPB |  |  | MDGA2 |
| NELL2 |  |  | SLC4A4 |
| HIPK3 |  |  | PBRM1 |
| SLC6A6 |  |  | LRP6 |
| ATRX |  |  | UGCG |
| PRKAR2A |  |  | DDX3X |
| XPA |  |  | PROX1 |
| ATP2B2 |  |  | PDE1A |
| GRM5 |  |  | ABLIM1 |
| SEMA3A |  |  | HSP90B1 |
| RNF5 (includes EG:6048) |  |  | COQ7 |
| B4GALT6 |  |  | PDLIM5 |
| BBX |  |  | ZBTB4 |
| SYNJ1 |  |  | CIT |
| ATCAY |  |  | CLMN |
| CPEB1 |  |  | EYA4 |
| PAFAH1B1 |  |  | NAPB |
| PQLC1 |  |  | ESCO1 |
|  |  |  | NELL2 |
|  |  |  | HIPK3 |
|  |  |  | MAPRE1 |
|  |  |  | SLC6A6 |
|  |  |  | ATRX |
|  |  |  | PRKAR2A |
|  |  |  | CSGALNACT1 |
|  |  |  | XPA |
|  |  |  | ATP2B2 |
|  |  |  | ZNF148 |
|  |  |  | GRM5 |
|  |  |  | SEMA3A |
|  |  |  | B4GALT6 |
|  |  |  | RNF5 (includes EG:6048) |
|  |  |  | SYNJ1 |
|  |  |  | BBX |
|  |  |  | ZNF622 |
|  |  |  | EIF4A1 |
|  |  |  | ATCAY |
|  |  |  | NRIP1 |
|  |  |  | PAFAH1B1 |
|  |  |  | CPEB1 |
|  |  |  | AKAP9 |
|  |  |  | PQLC1 |
